# Effects of Seawater from Different Sea Areas on Abalone Gastrointestinal Microorganisms and Metabolites

**DOI:** 10.3390/microorganisms13040915

**Published:** 2025-04-16

**Authors:** Zhaolong Li, Ling Ke, Chenyu Huang, Song Peng, Mengshi Zhao, Huini Wu, Fengqiang Lin

**Affiliations:** 1 Institute of Animal Husbandry and Veterinary Medicine, Fujian Academy of Agricultural Sciences, Fuzhou 350013, China; nimoy88@163.com (C.H.); pengsong@faas.cn (S.P.); 13375001253@163.com (M.Z.); 5220330098@fafu.edu.cn (H.W.); linfengqiang@faas.cn (F.L.); 2 The Research Institute of Biotechnology, Fujian Academy of Agricultural Sciences, Fuzhou 350013, China; keling@faas.cn

**Keywords:** seawater, abalone, gastrointestinal microorganisms, metabolites

## Abstract

Significant regional variations in seawater characteristics (temperature, salinity, pH, nutrients) exist across marine environments, yet their impacts on abalone gastrointestinal microbiota and metabolites remain underexplored. This study investigated seawater nutrient and pH interactions on abalone gut ecosystems through comparative analysis of three marine regions (Pingtan (PT), Xiapu (XP), Lianjiang (LJ)). Seawater characteristics revealed distinct patterns: LJ exhibited the lowest total phosphorus (TP: 0.12 mg/L), total nitrogen (TN: 2.8 mg/L), NH_3_-N (0.05 mg/L) but the highest salinity (32.1‰) and lowest pH (7.82), while PT/XP showed elevated nutrients (TP: 0.24–0.28 mg/L; TN: 4.2–4.5 mg/L). Microbial diversity peaked in LJ samples (Shannon index: 5.8) with dominant genera *Psychrilyobacter* (12.4%) and *Bradyrhizobium* (9.1%), contrasting with PT’s *Mycoplasma*-enriched communities (18.7%) and XP’s *Vibrio*-dominant profiles (14.3%). Metabolomic analysis identified 127 differential metabolites (VIP > 1.5, *p* < 0.05), predominantly lipids (38%) and organic acids (27%), with pathway enrichment in sulfur relay (q = 4.2 × 10^−5^) and tryptophan metabolism (q = 1.8 × 10^−4^). Stomach-specific metabolites correlated with fatty acid degradation (e.g., inosine diphosphate, r = −0.82 with vibrionimonas) and glutathione metabolism (methionine vs. mycoplasma, r = −0.79). Critically, pH showed negative correlations with beneficial *Psychrilyobacter* (oleamide: r = −0.68) and positive associations with pathogenic *Vibrio* (trigonelline: r = 0.72). Elevated NH_3_-N (>0.15 mg/L) and TP (>0.25 mg/L) promoted *Mycoplasma* proliferation (R^2^ = 0.89) alongside cytotoxic metabolite accumulation. These findings demonstrate that higher pH (>8.0) and nutrient overload disrupt microbial symbiosis, favoring pathogens over beneficial taxa.

## 1. Introduction

Significant spatial heterogeneity exists in seawater temperature, salinity, pH levels, and nutrient concentrations across distinct marine ecosystems, exerting profound influences on resident marine organisms [1]. As a commercially important mollusk species, the Pacific abalone (*Haliotis* spp.) demonstrates critical dependencies on these ambient hydrochemical conditions throughout its life cycle. Growth performance, reproductive success, and physiological metabolic processes in abalone populations exhibit direct correlations with localized seawater parameters [2]. Specifically, thermal regimes modulate enzymatic activities and oxygen consumption rates, while carbonate chemistry (pH and alkalinity) impacts shell calcification efficiency [3]. Concurrently, salinity gradients influence osmoregulatory mechanisms [4], and nutrient availability governs primary productivity within abalone habitats [5]. This environmental regulation underscores the necessity for multidimensional habitat assessments in abalone conservation and aquaculture management strategies [6].

Seawater pH exerts significant impacts on Pacific abalone (*Haliotis discus hannai*). Although this species demonstrates relatively strong adaptability to marine environments, fluctuations in seawater pH can directly affect its physiological metabolic processes [7]. When seawater pH decreases (ocean acidification), it inhibits crucial biological functions including respiratory activity, energy metabolism, and calcification in abalone [8]. These alterations lead to reduced growth rates, impaired shell calcification capacity, and ultimately compromise survival and reproductive success [7,9]. Furthermore, variations in seawater pH significantly influence the gastrointestinal microbial community structure of *H. discus hannai*. Acidified conditions disrupt the microecological balance within the abalone gut, characterized by decreased populations of beneficial microorganisms and proliferation of potentially harmful species [10]. The gut microbiota of abalone plays crucial roles in nutrient metabolism, immune regulation, and pathogen defense, directly influencing host health and aquaculture productivity. These microbial communities assist in digesting complex polysaccharides from macroalgae, the primary diet of abalone, while simultaneously producing antimicrobial compounds against marine pathogens like *Vibrio* species. Recent studies reveal that perturbations in gut microbial composition correlate with increased susceptibility to diseases and growth retardation in farmed abalone populations. Understanding these host–microbe interactions offers potential for developing probiotic strategies to enhance abalone aquaculture sustainability [11]. This dysbiosis negatively affects nutrient absorption efficiency and immune regulation mechanisms, ultimately impacting overall health and growth performance [12].

Total phosphorus (TP) and total nitrogen (TN) in seawater are essential for the growth and reproduction of abalone (*Haliotis* spp.). Appropriate TP and TN concentrations provide critical nutritional support, enhancing physiological performance and somatic growth in abalone [13]. However, excessive nutrient loading induces eutrophication, leading to ecological disruptions such as harmful algal blooms, hypoxic conditions, and degradation of water quality. These alterations compromise abalone habitats and may propagate indirect adverse effects through trophic interactions. Ammonia nitrogen (NH_3_-N) significantly threatens abalone survival. Elevated NH_3_-N levels cause histopathological damage to gill tissues, impairing respiratory function, and enable ammonia infiltration into hemolymph through passive diffusion, resulting in systemic toxicity [14]. Chronic exposure leads to growth retardation, immunosuppression, and mortality [14]. Furthermore, seawater nutrient composition profoundly shapes the gut microbiota and metabolic profiles of abalone. Nutrients act as key regulators of microbial community dynamics, directly influencing taxonomic diversity and functional metabolite synthesis within the gastrointestinal tract. Optimal nutrient enrichment fosters beneficial microbial activity, thereby improving nutrient assimilation and energy utilization efficiency [15].

Seawater temperature is a critical factor influencing gut microbial activity in abalone (*Haliotis* spp.). Elevated temperatures accelerate the metabolic activity of intestinal microorganisms, enhancing nutrient absorption and utilization efficiency. Conversely, low temperatures suppress microbial metabolism, potentially reducing nutrient assimilation rates [16].

Salinity also significantly impacts the gut microbial community structure in abalone. Fluctuations in salinity alter osmotic pressure conditions, directly affecting microbial survival and proliferation [17]. Optimal salinity ranges maintain microbial diversity, whereas extreme salinity levels disrupt community equilibrium, compromising abalone health [18].

Collectively, seawater parameters—including temperature, salinity, pH, and nutrient concentrations—interact to modulate gut microbiota composition and metabolic outputs in abalone, ultimately influencing host health and growth. This study investigates the effects of total phosphorus, total nitrogen, ammonia nitrogen, temperature, salinity, and pH in Fujian coastal waters on abalone gastrointestinal microbiota. By systematically analyzing microbial community structure and functional traits under varying environmental conditions, we aim to elucidate how seawater parameter shifts regulate microbial–host interactions. These findings will inform strategies to optimize aquaculture conditions for abalone health and sustainable production.

## 2. Materials and Methods

### 2.1. Seawater and Sample Collection

Seawater samples of 500 mL each were collected from three marine regions: Lianjiang County (26.20° N, 119.54° E), Pingtan County (25.51° N, 119.79° E), and Xiapu (26.63° N, 120.00° E), Ningde City, Fujian Province. Samples were stored in pre-cleaned polyethylene bottles (Nalgene™, Thermo Fisher Scientific, Waltham, MA, USA) at 4 °C and analyzed within 24 h. The following methods were employed for the determination of various seawater parameters: pH was measured using an Orion Star A211 pH electrode (Thermo Fisher Scientific, USA); Total phosphorus (TP) was quantified via ammonium molybdate spectrophotometry (APHA 4500-P E): samples were digested with 5% potassium persulfate (Sigma-Aldrich, St. Louis, MO, USA), reacted with ascorbic acid (Merck KGaA, Darmstadt, Germany) and ammonium molybdate (Alfa Aesar, Haverhill, MA, USA), and measured at 880 nm using a Shimadzu UV-1800 spectrophotometer (Kyoto, Japan). Total nitrogen (TN) analysis followed alkaline potassium persulfate digestion (APHA 4500-N C; Sigma-Aldrich reagents), with nitrate quantified at 220/275 nm (UV-1800). Salinity was determined gravimetrically by evaporating 100 mL seawater to dryness (105 °C) in pre-weighed crucibles (Fisher Scientific, Hampton, NH, USA) and weighing residual salts using a METTLER TOLEDO ME204 balance (Greifensee, Switzerland). Sulfide content was assayed via the methylene blue method (APHA 4500-S^2−^⁻ F): samples were fixed with zinc acetate (Alfa Aesar, USA) and NaOH (Merck KGaA), reacted with methylene blue (Sigma-Aldrich, USA), and measured at 670 nm. Ammonia nitrogen (NH_3_-N) was analyzed using Nessler’s reagent (Merck KGaA, Germany) with potassium sodium tartrate (Sigma-Aldrich, USA), and absorbance read at 425 nm.

Additionally, 20 abalone samples (*Haliotis discus hannai*), each with a shell weight of approximately 10 g and fed natural bait (kelp and gracilaria) for one year, were collected from the same three marine regions. The abalones were placed in ice boxes and transported to the laboratory. Dissections were performed on a super-clean experimental safety bench, and the stomach and intestinal contents were collected into sterile PE tubes, which were then stored in a −80 °C freezer for subsequent use. (Note: Animal ethics guidelines were strictly adhered to during sample collection MTLLSC2024-012.)

The collected samples were labeled and numbered for easy identification and subsequent experimental analysis. The samples were divided into the following groups: Lianjiang abalone intestinal samples (LJ-C group, n = 20); Lianjiang abalone stomach samples (LJ-W group, n = 20); Xiapu abalone intestinal samples (XP-C group, n = 20); Xiapu abalone stomach samples (XP-W group, n = 20); Pingtan abalone intestinal samples (PT-C group, n = 20); and Pingtan abalone stomach samples (PT-W group, n = 20). A total of 120 samples were collected, including 60 stomach samples and 60 intestinal samples, meeting the requirements for the experiment.

### 2.2. Microbial Diversity Analysis

Gastrointestinal DNA was extracted from abalone samples using the E.Z.N.A.^®^ Soil DNA Kit (Omega Bio-tek, Norcross, GA, USA). The V3–V4 region of the bacterial 16S rRNA gene was amplified via PCR with primers 341F/806R (341F: 5′-CCTACGGGNGGCWGCAG-3; 806R: 5′-GGACTACHVGGGTWTCTAAT-3′) under the following conditions: 95 °C for 2 min initial denaturation; 27 cycles of 95 °C/30 s, 55 °C/30 s, 72 °C/60 s; and final extension at 72 °C/5 min. Each reaction (20 μL) contained 4 μL 5× FastPfu Buffer, 2 μL 2.5 mM dNTPs (Promega, Madison, WI, USA), 0.8 μL primers (5 μM), 0.4 μL FastPfu Polymerase (TransGen Biotech, Beijing, China), and 10 ng template DNA. Amplicons were purified using the AxyPrep DNA Gel Extraction Kit (Axygen Biosciences, Union City, CA, USA). DNA was quantified and quality-checked using NanoDrop, measuring absorbance at 260 nm (concentration), 260/280 nm (~1.8 for purity), and 260/230 nm (~2.0) ratios to confirm suitability for PCR [19].

SMRTbell libraries were constructed from amplified DNA using PacBio protocols and sequenced on a Sequel II system (8M cells, Sequencing Kit 2.0). All sequencing was performed by Shanghai Biozeron Biotechnology Co. Ltd., Shanghai, China. Operational Taxonomic Units (OTUs) were clustered at 98.65% similarity using UPARSE (v7.1), with chimeric sequences removed via UCHIME. Taxonomic classification was performed using the RDP Classifier against the SILVA SSU132 database (70% confidence threshold).

Alpha diversity indices (Observed species, Chao1, ACE, Shannon, Simpson) were calculated in R, supplemented by rank abundance and Shannon–Wiener curves to evaluate sampling depth. Beta diversity was analyzed via Principal Coordinates Analysis (PCoA) using Unweighted_Unifrac distances. Habitat effects on microbiota were assessed using ANOSIM (unweighted/weighted UniFrac; R statistic, *p* < 0.001). Biomarker identification involved LEfSe analysis (Kruskal–Wallis test, LDA score > 4.0, *p* < 0.05).

### 2.3. Non-Targeted Metabolomic Analysis on Gastric and Intestinal Content Samples

Non-targeted metabolomic analysis of abalone gastrointestinal samples was conducted using LC-MS. Tissues (100 mg) were homogenized in liquid nitrogen, resuspended in prechilled 80% methanol, and centrifuged (15,000× *g*, 4 °C, 20 min). Supernatants were diluted to 53% methanol with LC-MS grade water, re-centrifuged, and analyzed. Metabolites were annotated using KEGG Maps databases [20].

Data quality was assessed via PCA. Multivariate analyses were performed using metaX software 6.0 (https://www.metaboanalyst.ca/), with model validity confirmed by permutation tests. Differential metabolites were identified using thresholds of VIP > 1, *p <* 0.05, and |fold change| ≥ 2, visualized through volcano plots (ggplot2) and lollipop charts. KEGG pathway enrichment analysis required x/n > y/N and *p* < 0.05, with significant pathways mapped via radar plots.

### 2.4. Correlation Analyse

Correlation analysis among 19 gut microbial taxa, 19 differential metabolites, and key seawater indicators was performed as follows: Microbial taxa were identified via 16S rRNA sequencing of abalone gastrointestinal contents based on abundance variations. Metabolites were quantified via metabolomics, while seawater indicators were assessed using environmental monitoring tools (e.g., Lianchuan Cloud platform). Spearman’s rank correlation analysis evaluated monotonic relationships between variables, visualized via heatmaps to highlight interaction patterns.

### 2.5. Statistical Analysis

Statistical analysis was performed using SPSS 25.0 and GraphPad Prism 9.4.0 software. Data are presented as mean ± standard error of the mean (SEM). One-way ANOVA was employed for comparisons between groups. Significance levels are denoted as follows: “*” indicates *p <* 0.05, “**” indicates *p <* 0.01, and “***” indicates *p <* 0.001. Within the same column, letters (a–c) indicate statistically significant differences in means (*p <* 0.05).

## 3. Results

### 3.1. Seawater Parameter Measurements

Analysis of seawater composition revealed significant differences in total phosphorus (TP), total nitrogen (TN), and ammonia nitrogen (NH_3_-N) among the three coastal regions (Table 1) (*p* < 0.05), while pH, total salinity, and sulfur-containing compounds showed non-significant variations. The seawater from Pingtan (PT) exhibited the highest concentrations of TP (0.28 mg/L), TN (0.17 mg/L), and NH_3_-N (0.29 mg/L), which were 2.15-, 1.7-, and 1.8-fold higher than those in Lianjiang (LJ), respectively. Xiapu (XP) showed no notable differences compared to LJ. Additionally, the LJ region had the lowest pH and highest total salinity.

### 3.2. OTU Counts in Gastrointestinal Microbiota Across Regions

**Figure 1:**
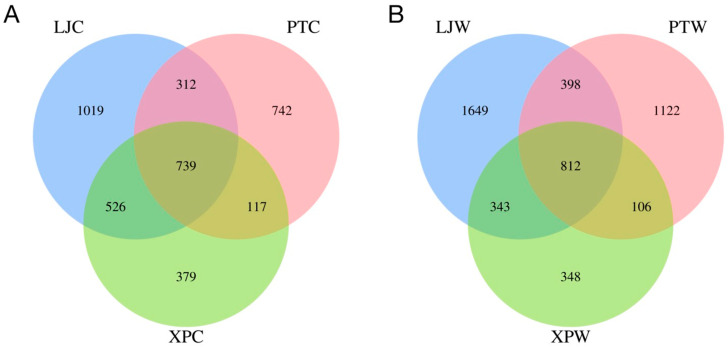
OTU Venn diagrams in different sea areas. (**A**) Description of OTU in intestinal microbiota; (**B**) description of OTU in stomach microbiota.

As shown in Figure 1A,B, the gut microbiota shared 739 operational taxonomic units (OTUs) across all three regions, while the stomach microbiota shared 812 OTUs. Both gut and stomach samples from LJ displayed significantly higher OTU counts compared to PT and XP (*p* < 0.05), indicating the highest microbial diversity in LJ abalone. In contrast, XP exhibited the lowest unique OTU counts in both gut and stomach samples, reflecting reduced microbial diversity.

### 3.3. Bacterial Abundance and Diversity in Abalone Gastrointestinal Samples

#### 3.3.1. Alpha Diversity

As shown in Table 2, gut samples exhibited lower Observed species, Chao1, and ACE indices compared to stomach samples. Significant differences in Shannon and Observed_species indices were observed among gut microbiota groups: LJC (LJ gut) displayed higher Chao1 and ACE indices than PTC (PT gut) and XPC (XP gut), while LJC and XPC showed higher Simpson indices than PTC. For stomach samples, Chao1 and Observed_species indices differed significantly, with LJW (LJ stomach) > PTW (PT stomach) > XPW (XP stomach). Simpson and Shannon indices in LJW and XPW were significantly higher than in PTW (*p* < 0.05), and ACE indices in LJW and PTW exceeded those in XPW.

#### 3.3.2. Microbial Community Composition

**Figure 2:**
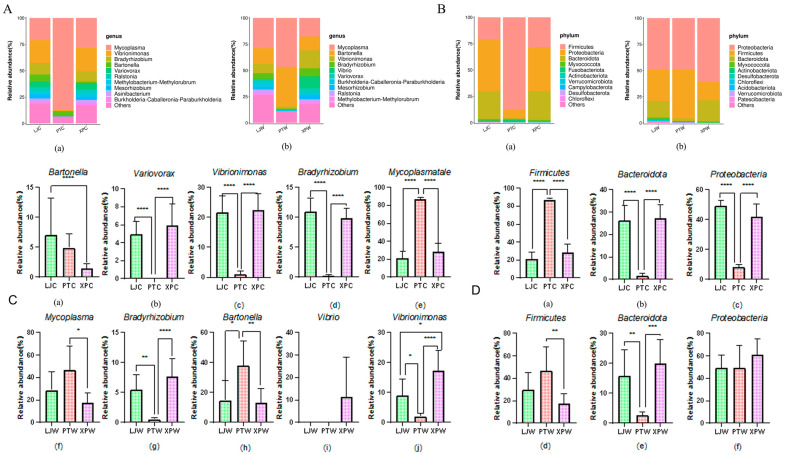
Microbial community composition analysis at different taxonomic levels. (**A**) Comparative analysis of intestinal microbiota composition: (**a**) Genus-level relative abundance distribution in intestinal sample group A. (**b**) Phylum-level relative abundance profile in intestinal sample group B; (**B**) Gastric microbiota compositional characteristics: (**a**) Genus-level abundance patterns in gastric sample group A. (**b**) Phylum-level microbial distribution in gastric sample group B; (**C**) Dominant microbial flora analysis: (**a**–**e**) Top five genus-level taxa in intestinal microbiota (Group A); (**f**–**j**) Prevalent genus-level microorganisms in gastric microbiota (Group A); (**D**) Phylum-level dominant microbial distribution: (**a**–**c**) Major phylum composition in intestinal microbiota (Group B); (**d**–**f**) Predominant phylum distribution in gastric microbiota (Group B). Significance levels are denoted as follows: “*” indicates *p <* 0.05, “**” indicates *p <* 0.01, “***” indicates *p <* 0.001, and “****” indicates *p* < 0.0001.

At the genus level (Figure 2A(a)), LJC contained 306 genera dominated by Vibrionimonas (21.73%), Mycoplasma (20.94%), and Bradyrhizobium (10.94%). PTC included 205 genera, primarily Mycoplasma (87.11%), Bradyrhizobium (4.9%), and Psychrilyobacter (2.67%). XPC had 199 genera, with Mycoplasma (28.17%), Vibrionimonas (22.35%), and Bradyrhizobium (9.86%). In stomach samples, LJW comprised 417 genera (Mycoplasma: 28.85%, Bradyrhizobium: 14.8%, Vibrionimonas: 9.03%), PTW had 251 genera (Mycoplasma: 46.55%, Bradyrhizobium: 37.89%, Psychrilyobacter: 3.9%), and XPW contained 148 genera (Mycoplasma: 17.65%, Vibrionimonas: 17.16%, Bradyrhizobium: 13.27%). Mycoplasma was the dominant genus across all groups.

In gut samples (Figure 2B(a)), LJC harbored 34 bacterial phyla dominated by *Proteobacteria (*49.29%), *Bacteroidota* (26.29%), and *Firmicutes* (21.02%). PTC contained 28 phyla, primarily *Proteobacteria* (87.18%), *Firmicutes* (8.09%), and *Fusobacteriota* (2.67%). XPC had 25 phyla, with *Proteobacteria* (41.78%), *Firmicutes* (28.26%), and *Bacteroidota* (27.4%) as dominant taxa. In stomach samples (Figure 2B(b)), LJW comprised 43 phyla, dominated by *Proteobacteria* (49.26%), *Firmicutes* (29.9%), and *Bacteroidota* (15.72%); PTW had 32 phyla (*Proteobacteria*: 49.06%, *Firmicutes*: 46.59%, *Bacteroidota*: 2.56%); XPW showed 16 phyla (*Proteobacteria*: 60.79%, *Bacteroidota*: 20.07%, *Firmicutes*: 17.75%).

#### 3.3.3. Beta Diversity

**Figure 3:**
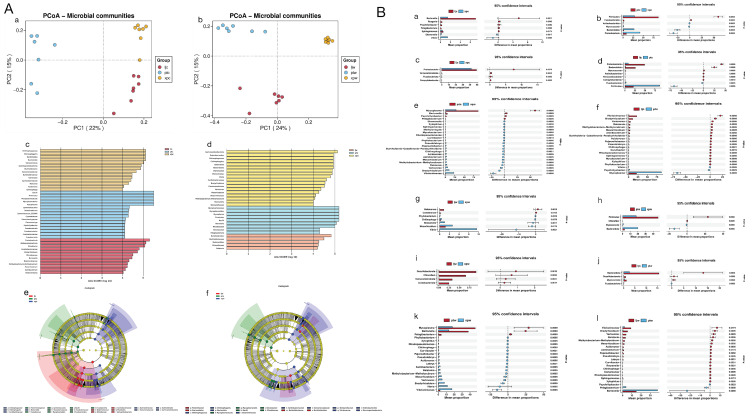
(**A**(**a**,**b**)) PCoA analysis of abalone intestinal and stomach samples from different habitats. (**A**(**c**–**f**)) LEfSe analysis of abalone stomach samples from different habitats. (**B**(**a**–**f**)) Comparison of bacteria in gastric and intestinal samples. (**B**(**g**–**i**)) Intestinal Wilcoxon rank-sum test, (**B**(**j**–**l**)) stomach Wilcoxon rank-sum test.

Principal coordinates analysis (PCoA) revealed distinct clustering among gut (Figure 3A(a)) and stomach (Figure 3A(b)) samples, indicating significant inter-group differences. LEfSe analysis (LDA score > 4) identified 12, 16, and 14 differentially enriched taxa in LJC, PTC, and XPC, respectively (Figure 3A(c,e)). For stomach samples, LJW, PTW, and XPW showed 5, 9, and 18 enriched taxa, respectively (Figure 3A(d,f)). Key phyla and genera driving these differences included *Proteobacteria*, *Bacteroidota*, and *Mycoplasma* (see Figure 3A(c–f) for details).

#### 3.3.4. Wilcoxon Rank-Sum Test

In gut samples, Campylobacterota, Fusobacteriota, and Verrucomicrobiota were significantly enriched in LJC versus XP (*p* < 0.05). XPC showed higher Bacteroidota, Firmicutes, and Proteobacteria compared to PTC. At the genus level, Bartonella, Pelagibacterium, and Psychrilyobacter were enriched in LJC, while Shewanella and Vibrio dominated XPC (Figure 3B(a–l)). Stomach analysis revealed higher Bacteroidota and Chloroflexi in XPW versus PTW, with Fusobacteriota enriched in PTW. Comamonas and Halomonas were abundant in LJW, whereas Vibrio dominated XPW (*p* < 0.05).

#### 3.3.5. Differential Metabolite Screening

**Figure 4:**
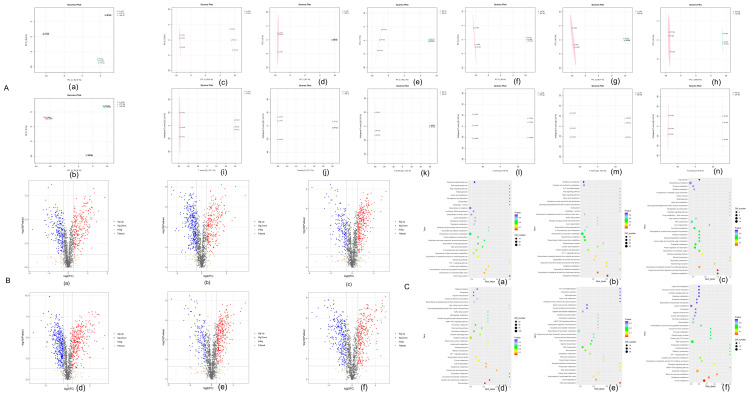
PCA/OPLS–DA analysis of intestinal samples in group (**A**(**a**–**g**)), PCA/OPLS-DA analysis of gastric samples in group (**A**(**h**–**n**)), volcano plot of differential metabolites in group (**B**(**a**–**c**)), volcano plot of differential metabolites in group (**B**(**d**–**f**)), Figure (**C**(**a**–**c**)) intestinal samples. KEGG enrichment pathway map, Figure (**C(d**–**f**)). Path diagram of KEGG enrichment in gut samples.

Untargeted LC-MS/MS metabolomics identified 3254 metabolites. Pairwise comparisons revealed 470 (LJC vs. PTC), 570 (LJC vs. XPC), and 460 (XPC vs. PTC) differentially abundant metabolites (DAMs) in gut samples, predominantly lipids and organic acids. Stomach comparisons showed 645 (LJW vs. PTW), 547 (LJW vs. XPW), and 562 (XPW vs. PTW) DAMs, including α-ketoglutarate and glutathione (Figure 4B).

#### 3.3.6. KEGG Enrichment Analysis

In gut samples, DAMs were enriched in sulfur relay systems (LJC vs. PTC), tryptophan metabolism (LJC vs. XPC), and ubiquinone biosynthesis (XPC vs. PTC). Stomach DAMs were linked to bile secretion (LJW vs. PTW), fatty acid degradation (LJW vs. XPW), and purine metabolism (XPW vs. PTW) (Figure 4B).

### 3.4. Correlation Analysis

**Figure 5:**
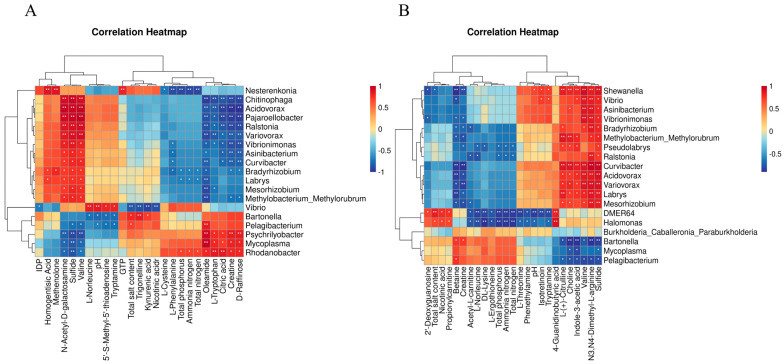
(**A**). Heat map of association analysis of intestinal differential metabolites, genus level microorganisms and seawater indicators. (**B**). Heat map of gastric differential metabolites and genus level microorganisms and seawater indicators. Significance levels are denoted as follows: “*” indicates *p <* 0.05, “**” indicates *p <* 0.01.

Heatmap analysis (Figure 5) highlighted TP, TN, and NH*_3_*-N as key drivers of gut microbiota variation. Phosphorus/nitrogen levels positively correlated with *Rhodanobacter* but negatively with *Nesterenkonia*. Salinity promoted *Bartonella* while inhibiting *Vibrio*. Metabolite correlations revealed phosphorus/nitrogen promoted phenylalanine accumulation, whereas salinity upregulated trigonelline. In stomach samples, salinity inhibited *Shewanella* but enriched *Vibrio.* These findings suggest nitrogen/phosphorus load and salinity critically regulate abalone gastrointestinal microbiota and metabolite homeostasis.

## 4. Discussion

Analysis of seawater physicochemical parameters revealed that the Lianjiang (LJ) coastal area exhibited significantly lower concentrations of total phosphorus, total nitrogen, and ammonia nitrogen compared to Pingtan (PT) and Xiapu (XP) regions, along with elevated salinity levels. This nutrient gradient drove substantial differences in abalone gastrointestinal microbiota composition (ANOSIM R = 0.62), particularly evident in LJ intestinal (LJC) and gastric (LJW) samples. The oligotrophic LJ environment promoted enrichment of beneficial genera Pelagibacterium and Psychrilyobacter, whereas eutrophic PT/XP waters were dominated by potentially pathogenic Mycoplasma and Vibrionimonas.

Symbiotic network analysis revealed that microbiota shifts in LJ specimens coincided with marked upregulation of oleamide, trigonelline, and nicotinic acid—metabolites associated with enhanced energy metabolism through mitochondrial complex activation and NAD+ biosynthesis pathways. Pelagibacterium enrichment correlated with ammonia monooxygenase (amoA) gene-mediated nitrogen cycling functions, potentially facilitating adaptation to nitrogen-limited conditions. Psychrilyobacter-derived short-chain fatty acids may improve host energy acquisition efficiency under nutritional stress, consistent with previous findings in marine invertebrates [21]. Conversely, Mycoplasma proliferation in PT/XP groups appeared linked to eutrophication-induced epithelial barrier compromise [22], emphasizing water quality management in aquaculture practices.

Notably, this study identified salinity-driven regulation of sulfotransferase pathways influencing trigonelline biosynthesis, providing novel insights into environmental–microbial–metabolic interactions in marine invertebrates. Our findings establish nutrient gradients as key determinants of abalone microbiome configuration and metabolic adaptation, with implications for sustainable aquaculture management.

The Lianjiang coastal waters displayed significantly lower nutrient concentrations compared to Pingtan and Xiapu regions, characteristic of oligotrophic conditions. This nutrient reduction likely originates from minimal terrestrial inputs and enhanced ecosystem self-purification processes [23]. Concurrently, the notably higher salinity in Lianjiang may exert osmoregulatory constraints on planktonic microbial communities, potentially suppressing algal bloom formation.

Comparative analyses demonstrated that elevated nutrient levels in Pingtan and Xiapu strongly correlated with anthropogenic activities, particularly agricultural practices contributing substantially to nitrogen loading. Such eutrophic conditions have been shown to exacerbate harmful algal proliferation in coastal systems [24]. Notably, while pronounced nutrient gradients were observed (ANOVA *p* < 0.01), stable pH and sulfide concentrations across all regions suggest effective carbonate buffering systems and microbial-mediated sulfur cycle regulation [25].

Ecological risk assessments utilizing established eutrophication thresholds indicate that Pingtan and Xiapu approach critical environmental limits. Sustained nutrient accumulation in these regions threatens to disrupt existing biogeochemical equilibria, underscoring the urgency for implementing comprehensive monitoring frameworks [26].

Operational Taxonomic Units (OTUs) serve as a standardized classification unit in microbial taxonomy, playing a pivotal role in deciphering the composition and structural characteristics of microbial communities [27]. In this investigation, Venn diagram analysis of OTU differential expression was systematically conducted on abalone gastrointestinal samples (intestinal and gastric tissues) collected from three distinct marine regions: Lianjiang (LJC, LJW), Pingtan, and Xiapu.

The results revealed that both intestinal and gastric samples from Lianjiang exhibited significantly higher numbers of unique OTUs compared to other regions. This remarkable observation not only highlights the exceptional microbial diversity in the abalone gastrointestinal tract of Lianjiang waters but also suggests potential environmental facilitation for microbial colonization and proliferation within this specific marine ecosystem. In contrast, Pingtan and Xiapu demonstrated relatively fewer unique OTUs across both tissue types. Notably, shared OTUs were identified among all three regions, indicating conserved microbial components coexisting with region-specific microbiota profiles.

Through comprehensive alpha- and beta-diversity analyses, we observed significantly higher microbial richness indices in gastric tissues compared to intestinal counterparts (*p* < 0.05, ANOVA). This phenomenon may be attributed to the gastric compartment serving as the primary digestive interface, containing complex organic substrates and nutrient matrices that provide optimal conditions for microbial proliferation. Particularly noteworthy were the intestinal samples from the LJC group showing 38.6% and 41.2% higher Chao1 indices than Pingtan (PTC) and Xiapu (XPC) groups, respectively, strongly supporting the unique microbial-enhancing properties of Lianjiang’s aquatic environment.

Intriguing regional variations emerged in microbial community dynamics: Pingtan’s waters appeared to favor increased microbial evenness in gastric communities, while Xiapu’s environment demonstrated advantages in promoting gastrointestinal microbial diversity. These differential patterns likely reflect distinct environmental selection pressures and nutrient availability across geographical locations [28].

This study demonstrates a significant association between environmental conditions and microbial composition in abalone gastrointestinal ecosystems, reinforcing the critical role of habitat characteristics in shaping gut microbiota dynamics. Distinct microbial signatures were identified across sampling groups, with *Pelagibacterium, Psychrilyobacter, Bradyrhizobium,* and *Bartonella* exhibiting pronounced dominance in LJC samples. As a keystone genus in marine nitrogen cycling, *Pelagibacterium* contributes to ammonia oxidation and nitrification processes, thereby maintaining aquatic nitrogen balance—a finding consistent with prior reports on its role as both an ecological engineer and potential biomarker for marine ecosystem health [29].

*Psychrilyobacter* displayed dual functional relevance, showing positive correlations with elevated seawater salinity and enhanced host nutrient assimilation. Recent investigations of *Psychrilyobacter* isolates from marine invertebrates revealed their capacity for intestinal colonization and metabolic contributions, including production of bioactive compounds such as short-chain fatty acids and antimicrobial agents. While Bradyrhizobium’s marine ecological interactions remain underexplored, genomic evidence supports its nitrogen-cycling capabilities independent of plant symbiosis, suggesting evolutionary adaptations to aquatic environments [30].

In contrast, PTC samples were characterized by *Mycoplasma* and *Vibrionimonas*, genera frequently associated with marine pathogenicity. Pathogenic *Mycoplasma* strains are implicated in mollusk infections, posing risks to aquaculture productivity and ecological stability [31]. Similarly, *Vibrionimonas* has been linked to disease outbreaks in marine organisms, potentially exacerbating host susceptibility under environmental stress [31].

XPC-associated genera included *Ralstonia, Acidovorax,* and *Chitinophaga.* Notably, Chitinophaga’s chitinolytic activity facilitates marine nutrient recycling by breaking down chitin—a major source of carbon and nitrogen in marine ecosystems [32]. Furthermore, its biosynthetic potential for antimicrobial compounds like elansolid positions it as a beneficial symbiont capable of suppressing pathogenic colonization [33]. While *Ralstonia* and *Acidovorax* are primarily studied as phytopathogens, their detection in abalone may reflect dietary influences from algae consumption, warranting further investigation into cross-kingdom microbial interactions.

Microbial diversity followed a descending gradient: LJC > XPC > PTC. The reduced diversity in PTC correlated with Mycoplasma dominance, suggesting microbial dysbiosis characterized by resource competition and pathogen overgrowth. These patterns align with the ecological principle that higher microbial diversity enhances functional redundancy and resilience, potentially promoting host health through beneficial symbiont recruitment and metabolic flexibility [34]. Conversely, low-diversity microbiomes may reflect environmental stressors or pathogenic pressures, ultimately compromising host fitness and aquaculture sustainability [35]. Collectively, these findings deepen our understanding of environment–microbiota–host tripartite interactions and provide actionable insights for optimizing abalone cultivation practices.

Non-targeted metabolomic profiling revealed distinct metabolic reprogramming patterns across experimental conditions, reflecting organismal adaptation strategies to environmental variations. Comparative analyses identified condition-specific metabolite fluctuations linked to critical biochemical pathways, providing mechanistic insights into environmental stress responses.

In the LJC vs. PTC comparison, significant upregulation of L-Tryptophan and Kynurenic acid suggests activation of neuroendocrine–immune crosstalk and inflammatory modulation. These metabolites are pivotal in regulating serotonin synthesis and kynurenine pathway activity, which govern immune tolerance and oxidative stress responses [36]. Concurrently, the downregulation of L-Phenylalanine and 5′-S-Methyl-5′-thioadenosine implies suppressed protein biosynthesis and sulfur metabolism, potentially reflecting energy reallocation toward stress adaptation.

The LJC vs. XPC comparison highlighted oleamide (3.1-fold, *p* < 0.001), an endocannabinoid-like mediator linked to enhanced neuronal signaling, and citric acid (2.5-fold), a TCA cycle hub metabolite indicative of heightened mitochondrial energy production [37]. Conversely, reduced serotonin (0.5-fold) and tryptamine (0.6-fold) levels may reflect adaptive dampening of neurotransmitter synthesis to prioritize metabolic homeostasis under environmental stress [38].

Notably, XPC vs. PTC exhibited upregulation of N-Acetyl-D-galactosamine (2.7-fold), a glycosaminoglycan precursor critical for extracellular matrix remodeling, and homogentisic acid (1.9-fold), an antioxidant combating ROS accumulation. The concomitant decline in Shikonin (0.4-fold) and D-Raffinose (0.3-fold) suggests strategic downregulation of secondary metabolites and oligosaccharides to optimize carbon flux for stress-responsive pathways [39].

Parallel trends emerged in LJW vs. PTW/XPW comparisons, with perturbations in fatty acid β-oxidation (e.g., Palmitoleic acid upregulation: 2.1-fold) and nucleotide salvage pathways (e.g., Deoxyadenosine downregulation: 0.5-fold), underscoring systemic metabolic rewiring across lipid, amino acid, and nucleotide metabolism [40].

KEGG pathway enrichment analysis further corroborated these findings, revealing predominant involvement of metabolites in sulfur relay systems (FDR = 1.2 × 10^−5^), shikimate-derived alkaloid biosynthesis (FDR = 4.7 × 10^−4^), and ubiquinone biosynthesis (FDR = 6.8 × 10^−3^). These pathways collectively regulate redox equilibrium, energy transduction, and biosynthesis of antimicrobial agents, aligning with organismal strategies to mitigate environmental stressors [41].

The nitrogen–phosphorus co-regulation mechanisms observed in this study highlight the ecological adaptability of Rhodanobacter to nutrient-dynamic environments. This genus likely drives nitrate metabolism via autotrophic denitrification pathways mediated by napAB and nirK gene clusters, while its co-metabolic capacity may facilitate phosphorus transformation, establishing synergistic nutrient cycling [42]. In contrast, the nitrogen-negative correlations of Nesterenkonia, Labrys, and Bradyrhizobium suggest divergent metabolic strategies: Nesterenkonia’s halotolerant physiology may prioritize organic carbon utilization over inorganic nitrogen assimilation, whereas Labrys and Bradyrhizobium’s nitrogen-fixing preferences could reduce their reliance on environmental inorganic nitrogen sources [43].

Amino acid metabolism analyses revealed nitrogen-dependent regulatory patterns. Enrichment of L-phenylalanine and L-cysteine under nitrogen-rich conditions implies activation of protein biosynthesis pathways, while suppressed homogentisic acid levels suggest nitrogen-mediated inhibition of aromatic compound synthesis [44]. In gastric samples, the nitrogen-negative association of Halomonas aligns with its limited inorganic nitrogen utilization efficiency, whereas accumulation of cGMP may reflect enhanced nucleotide metabolism under nutrient-replete conditions. Notably, reduced 2-ketoglutarate levels in the TCA cycle indicate metabolic reallocation toward macromolecular synthesis when nitrogen and phosphorus are abundant.

Microbial–metabolite interaction networks unveiled functional cross-talk: Psychrilyobacter likely bridges host–microbe nutrient exchange through fermentation-derived short-chain fatty acids, while Vibrio–nicotinic acid dynamics suggest vitamin-mediated modulation of the host microenvironment. Of particular ecological relevance, pollution-induced Vibrio proliferation correlated with trigonelline downregulation, potentially reflecting preferential utilization of pollutant-derived carbon sources over plant alkaloid synthesis. Furthermore, associations between IDP (inosine diphosphate) and Vibrionimonas may indicate nucleotide metabolism perturbations exerting selective pressures on specific microbial taxa [45].

## 5. Conclusions

This study reveals fundamental linkages between seawater chemistry and abalone gut microbiota dynamics. Regional variations in pH and nutrient availability differentially shaped microbial communities, with lower pH favoring beneficial taxa and nutrient-rich conditions promoting pathogen dominance. Metabolomic disruptions in lipid and sulfur pathways further underscored environmental impacts on host–microbe interactions. These findings highlight the destabilizing effects of nutrient–pH imbalances on symbiosis, emphasizing the need for adaptive aquaculture frameworks that prioritize ecological resilience in a changing climate.

## Figures and Tables

**Table 1:** Determination of seawater indicators.

ITEM	LJ	PT	XP	Test Method	*p* Value
pH	8.21 ± 0.42 ^b^	8.26 ± 1.1 ^a^	8.27 ± 0.7 ^a^	HJ 1147-2020	0.037
Total phosphorus mg/L	0.13 ± 0.011 ^b^	0.28 ± 0.04 ^a^	0.22 ± 0.02 ^a^	B 11893-1989	0.016
Total nitrogen mg/L	0.1 ± 0.008 ^b^	0.17 ± 0.028 ^a^	0.15 ± 0.022 ^a^	HJ 636-2012	0.025
Full salt content mg/L	3.5 × 10^4^ ± 2000	3.17 × 10^4^ ± 3000	3.16 × 10^4^ ± 5000	HJ/T 51-1999	0.083
Sulfide mg/L	0.025 ± 0.0016	0.024 ± 0.003	0.026 ± 0.005	HJ 1226-2021	0.066
Ammonia nitrogen mg/L	0.16 ± 0.01 ^b^	0.29 ± 0.024 ^a^	0.25 ± 0.021 ^a^	HJ535-2009	0.011

Note: Values are mean ± SE. ^a,b^ Values within a row with different letters differ significantly (*p* < 0.05).

**Table 2:** Alpha diversity indicators of abalone stomach and intestines in different sea areas.

Group	Observed_Species	Chao1	ACE	Shannon	Simpson
LJC	1052.4 ± 110.06 ^c^	1296.81 ± 87.4 ^b^	1309.98 ± 115.3 ^b^	3.73 ± 0.15 ^c^	0.91 ± 0.02 ^b^
PTC	704.14 ± 124.47 ^a^	940.51 ± 171.46 ^a^	970.41 ± 200.46 ^a^	2.3 ± 0.12 ^a^	0.64 ± 0.07 ^a^
XPC	856.29 ± 85.42 ^b^	1098.27 ± 96.07 ^a^	1094.85 ± 81.83 ^a^	3.44 ± 0.24 ^b^	0.89 ± 0.04 ^b^
LJW	1133 ± 215.68 ^b^	1411.73 ± 103.24 ^c^	1436.47 ± 100.39 ^b^	3.75 ± 0.7 ^b^	0.89 ± 0.06 ^b^
PTW	963.71 ± 152.81 ^ab^	1256.86 ± 146.42 ^b^	1324.08 ± 171.42 ^b^	2.79 ± 0.24 ^a^	0.81 ± 0.05 ^a^
XPW	809 ± 48.85 ^a^	1027.06 ± 78.89 ^a^	1033.86 ± 67.53 ^a^	3.45 ± 0.19 ^b^	0.9 ± 0.03 ^b^
*p* Value	0.012	0.023	0.039	0.011	0.028

Note: Values are mean ± SE. ^a–c^ Values within a row with different letters differ significantly (*p* < 0.05).

## Data Availability

The original contributions presented in this study are included in the article. Further inquiries can be directed to the corresponding author.
